# Clozapine induced pneumonia: A case report of diagnostic difficulties in the time of Covid-19

**DOI:** 10.1192/j.eurpsy.2022.1839

**Published:** 2022-09-01

**Authors:** R. Softic, A. Tahirovic, G. Sulejmanpasic, A. Memic Serdarevic, A. Cesir, N. Becarevic

**Affiliations:** 1 University Clinical Center Tuzla, Of Psychiatry, Tuzla, Bosnia and Herzegovina; 2 Clinical Centre University of Sarajevo, Psychiatric Clinic, Sarajevo, Bosnia and Herzegovina

**Keywords:** Side effects, clozapine, Pneumonia

## Abstract

**Introduction:**

Clozapine is a drug that can cause several side effects. Among the less commonly described is a drug-induced lung disease. Due to its non-specific clinical presentation, it represents a diagnostic challenge. The diagnosis is made based on: 1. Association of exposure to the agent and development of symptoms, 2. Pulmonary infiltration, 3. Exclusion of other causes, 4. Withdrawal of symptoms when the agent is excluded from therapy. To date, there have been only a few descriptions of this condition.

**Objectives:**

Case report of rare side effect of clozapine.

**Methods:**

Case report

**Results:**

Case report: male patient (37) with schizophrenia, was hospitalized after a brutal suicide attempt. The PCR test for COVID-19 that was routinely performed on admission was negative. After the introduction of clozapine into therapy, the patient became febrile. There was a drop in oxygen saturation, a Lung CT scan showed inflammatory changes *(„ground-glass opacities“),* and COVID-19 pneumonia was suspected. Due to the worsening of the mental state, the dose of clozapine was increased. The physical condition further deteriorated: febrile, sO2 declining. After repeated PCR tests for COVID-19 (all negative), interstitial pneumonia caused by clozapine was suspected, and clozapine was excluded from therapy. The physical condition started to improve. Quetiapine was introduced, and occasional episodes of agitation were relieved with intramuscular diazepam. In the following days, the patient’s mental state improved and he was discharged.

**Conclusions:**

Despite its superiority over other antipsychotics, clozapine was with good rationale ranked third in treatment guidelines for schizophrenia.

**Disclosure:**

No significant relationships.

